# Compared to plasma, bronchial washing fluid shows higher diagnostic yields for detecting EGFR-TKI sensitizing mutations by ddPCR in lung cancer

**DOI:** 10.1186/s12931-020-01408-x

**Published:** 2020-06-09

**Authors:** Sang Hoon Lee, Eun Young Kim, Taehee Kim, Yoon Soo Chang

**Affiliations:** Department of Internal Medicine, Yonsei University College of Medicine, Seoul, Republic of Korea

**Keywords:** Bronchial washing fluid, Plasma, ddPCR, mEGFR, Lung cancer

## Abstract

**Background:**

The rate of diagnosis of advanced lung adenocarcinoma must be improved. In this study, we compared the detection rates of EGFR-tyrosine kinase inhibitor-sensitizing mutations (mEGFRs) in bronchial washing fluid (BWF) and the plasma of patients with lung adenocarcinoma using the tissue genotype as the standard reference.

**Methods:**

Paired blood and BWF specimens were collected from 73 patients with lung cancer. The tumor EGFR mutation status was determined by genotyping of the plasma and BWF samples using droplet digital PCR (ddPCR).

**Results:**

The study cohort included 26, 10, 10, and 27 patients with stage I, II, III, and IV disease. Of the 73 cases, 35 had a wild-type EGFR, and 19 had the L858R substitution and exon 19 deletion mutations. The areas under the receiver operator characteristic curves for sensitivity vs. specificity of ddPCR were 0.895 [95% confidence interval (CI): 0.822–0.969] for BWF and 0.686 (95% CI: 0.592–0.780) for plasma (*p* < 0.001). The fractional abundance was higher in BWF of the mEGFR-positive cases than in the plasma (*p* = 0.004), facilitating easy threshold setting and discrimination between mEGFR-positive and negative cases. When genotyping results obtained using plasma and BWF were compared for early lung cancer (stages I–IIIA), the diagnostic yields were significantly higher for BWF ddPCR, and the same tendency was observed for the advanced stages, suggesting that the BWF data may reflect the genotype status in early-stage patients.

**Conclusions:**

The mEGFR genotyping results obtained using BWF showed a higher diagnostic efficacy than did those obtained using the plasma. Thus, BWF-based genotyping may be a useful substitute for that using plasma in lung cancer.

## Background

In 2018, approximately two million people were newly diagnosed with lung cancer worldwide, and 1.76 million died of this devastating disease, accounting for 18.4% of all cancer-related deaths [1, 2]. Because the associated symptoms are not initially detectable and are similar to those of other respiratory diseases, lung cancer is typically diagnosed in advanced stages, when the disease cannot be cured by surgical resection. Thus, lung cancer is mainly treated with chemotherapy, targeted agents, and immune checkpoint inhibitors, based on biomarkers, along with radiotherapy.

Owing to recent advances in translational research, the outcomes and quality of life of patients with lung cancer have greatly improved through the use of targeted therapy, including EGFR-, ALK-, and ROS1-targeting agents, compared with those of patients on conventional treatments, such as chemotherapy [3]. In a landmark placebo-controlled study, Iressa Survival Evaluation in Lung Cancer (ISEL), of advanced non-small cell lung cancer (NSCLC) refractory to previous chemotherapy, the EGFR-tyrosine kinase inhibitor (TKI) showed an efficacy in a subset of the study population, suggesting the need for biomarkers to predict therapeutic responses [4]. The ISEL-associated IPASS trial, which identified an EGFR-activating mutation [5], showed that the EGFR-TKI was more effective in patients with lung adenocarcinoma, who had EGFR-TKI-sensitizing mutations (mEGFRs) [6]. Further phase III trials, which compared the outcomes of first- or second-line EGFR-TKI treatments with those of platinum doublets, confirmed the beneficial effects of mEGFRs on the progression-free survival and response rates in patients with lung adenocarcinoma [7]. Therefore, it is important to identify target genes and accurately manage lung cancer, and mEGFRs are considered strong biomarkers for predicting the response to an EGFR-TKI.

Tissue biopsy specimens are used as part of the standardized protocol to detect EGFR target mutations. However, tissue biopsy is an invasive procedure, or it is impossible to obtain tissue biopsy samples, depending on the patient’s condition, tumor location, and size. Based on the flexible bronchoscopy biopsy results, the sensitivity of lung cancer diagnosis was 34% for peripheral tumors with a diameter < 2 cm and 63% for peripheral lesions with a diameter > 2 cm [8]; these suboptimal diagnostic yields make the identification of molecular biomarkers difficult. By contrast, 82.9% of specimens obtained by percutaneous core needle biopsies are appropriate for molecular marker analysis, with only 15.3% of the subjects experiencing pneumothorax and 9.4% showing complications after chest tube insertion [9].

Liquid biopsy using plasma is a simple, easy-to-repeat, and less invasive method, which may overcome the disadvantages and limitations of tissue biopsy. However, this method exhibits some disadvantages, as different assay platforms show different sensitivities and specificities and are based on different analytical approaches [10]. Furthermore, circulating tumor DNA (ctDNA) only constitutes 0.1–1.0% of cell-free DNA (cfDNA) in the plasma, and the half-life of ctDNA is approximately 90 min. Thus, the results obtained using the plasma are less accurate than those obtained by conventional tissue biopsy. By-products from the flexible bronchoscopy procedure, such as bronchial washing fluid (BWF) and/or bronchoalveolar lavage fluid (BALF), may be a useful alternative to biopsy specimens. Bronchoscopy is routinely performed in patients with suspected lung cancer, and BALF is a specific material that can reflect characteristics of the lung compartment [11]. Carvalho et al. [12] performed mass spectrometry of BALF from 90 patients with suspected lung cancer and found significantly different biomarkers between the lung cancer and non-lung cancer groups. These studies suggest that the use of BALF may be more effective than that of plasma for diagnosing lung cancer.

Droplet digital PCR (ddPCR) is an advanced technique, which shows high sensitivity and specificity for the detection of genetic alterations in ctDNA. Using a microfluidic chip system, ddPCR generates up to 20,000 droplets, which can be used to separate particular DNA fragments [13, 14].

Herein, we compared the performance of the ddPCR platform for the detection of mEGFRs in lung adenocarcinoma using plasma, which is a standard for liquid biopsy, and BWF, obtained during routine bronchoscopy.

## Methods

### Patients and clinical specimens

Paired blood and BWF specimens were collected from 73 patients with NSCLC between June 2016 and May 2019. The inclusion criteria were as follows: (1) pathologically proven NSCLC; (2) the tumor EGFR mutation (NM_005228.5) status determined by genotyping of tumor tissue, which was obtained simultaneously with blood and BWF samples; and (3) informed consent signed for the collection and use of BWF and blood samples. Patients with rare mEGFRs were excluded from the study. This study was performed in accordance with the amended Declaration of Helsinki and was approved by the independent hospital institutional review board (approval no. 3–2016-0225 and 3–2017-0321).

BWF and blood samples were obtained at the time of the initial visit for pathologic examination of lung tumor, and the interval for securing paired specimens was less than 24 h. Bronchoscopy was performed through the mouth after sedation of the patient with midazolam and fentanyl, and bronchial washing was performed using approximately 20 mL of sterile 0.9% saline by wedging the bronchoscope at a lung cancer site. BWF was collected as a residue after sending the tissue specimen for routine cytologic examination and microbial study. If the obtained BWF specimen was less than 5 mL, an additional specimen was obtained by bronchial washing once or twice and processed within 3 h of collection by centrifugation at 1800×*g* for 10 min at 4 °C. The supernatant was stored at − 80 °C until analysis. Seven milliliters of blood were collected in a Streck tube (Streck, La Vista, NE, USA); the sample was transferred to the laboratory within 8 h of collection and centrifuged at 1800×*g* for 10 min at 4 °C to obtain plasma, and the plasma was stored at − 80 °C. DNA was extracted from the plasma using the QIAamp circulating nucleic acid kit (Qiagen, Hilden, Germany) according to the manufacturer’s instructions.

### Droplet digital PCR

ddPCR was performed according to the manufacturer’s instructions (Bio-Rad, Hercules, CA, USA). mEGFRs were detected using probes (Bio-Rad) for the E19del (c.2235del15; p.E746_A750del) and L858R mutation (c.2573 T > G; p.Leu858Arg). A549 cells were used as a negative control. As positive controls, SNU1330 cells, harboring an EGFR E19del mutation (homozygote), and H1975 cells, containing the L858R and T790M mutations, were used in each experiment.

Droplets were generated using a QX100 droplet generator (Bio-Rad), and PCR amplification was performed using a thermal cycler (Bio-Rad). After PCR, droplets were streamed in a single file on a QX200 droplet reader (Bio-Rad) to count fluorescence-positive and fluorescence-negative droplets. Data were processed using the QuantaSoft software (Bio-Rad). The thresholds for the ddPCR results were determined using QuantaSoft and then manually inspected for further validation. The ddPCR results were considered to pass quality control when the number of droplets was more than 9000 and the wild-type gene sequence was present at more than 100 copies/mL [12]. Only data that passed initial quality control were further analyzed. Positivity was defined as the fractional abundance (Fa) of ≥0.044% (sensitivity, 42.1%; specificity, 91.4%) for the plasma samples and ≥ 0.015% for the BWF samples.

### Statistical analysis

Categorical and continuous parameters were evaluated using a chi-squared test and an independent samples *t*-test, respectively. Spearman’s correlation was used to evaluate the relationships between two variables. Areas under the curves (AUCs) for sensitivity vs. specificity of plasma and BWF ddPCR were calculated and compared using the pROC and gmodels R packages, respectively. A *p*-value of less than 0.05 was considered significant. Statistical analyses were performed using SPSS version 25.0 (SPSS, Inc., Chicago, IL, USA) or the R statistical package ver. 3.5.3 (Institute for Statistics and Mathematics, Vienna, Austria; www.R-project.org).

## Results

### Demographic characteristics of the study population

Table 1 presents the characteristics of the enrolled cases. The mean age of the study population was 65.3 ± 9.8 years; 38 (52.1%) patients were males, and 35 (47.9%) patients were females. Twenty-eight (38.4%) patients had a history of smoking, and the mean lifetime smoking level was 12.6 ± 21.4 pack-year. Nearly all enrolled patients (89.0%) had adenocarcinoma, and one (1.4%) had pulmonary sarcomatoid carcinoma. The mean longest diameter of tumor was 3.5 ± 2.0 cm. Twenty-six patients had stage I cancer; 10 each had stage II and stage III cancer; and 27 had stage IV cancer. Of the 73 patients, 35 (47.9%) were found to have a wild-type EGFR, and 38 patients showed mutations in the EGFR-tyrosine kinase domain, of which 19 patients had the L858R substitution and 19 had E19del. Except the stage, other baseline characteristics did not significantly differ between patients with early (stages I–IIIA) and advanced (stages IIIB–IV) lung cancer.

**Table 1:** Baseline characteristics of the study population

	Total (*n* = 73)	Early stage of lung cancer (*n* = 38)	Advanced stage of lung cancer (*n* = 35)
Age (year)	65.3 ± 9.8	65.0 ± 8.1	65.7 ± 11.5
Sex (F/M)	35/38 (47.9/52.1)	20/18 (52.6/47.4)	15/20 (42.9/57.1)
Smoking status			
Never smoker	45 (61.6)	25 (65.8)	20 (57.1)
Former smoker	22 (30.1)	10 (26.3)	12 (34.3)
Current smoker	6 (8.2)	3 (7.9)	3 (8.6)
Smoking amount (pack-year)	12.6 ± 21.4	11.3 ± 20.0	14.1 ± 23.2
Tumor type			
Adenocarcinoma	65 (89.0)	32 (84.2)	33 (94.3)
Squamous cell carcinoma	7 (9.6)	5 (13.2)	2 (5.7)
Sarcomatoid carcinoma	1 (1.4)	1 (2.6)	–
Tumor size (cm)	3.5 ± 2.0	3.0 ± 2.0	3.9 ± 1.9
Lung cancer stage			
I/II/III/IV	26/10/10/27	26/10/2/−	−/−/8/27
EGFR genotyping			
Wild type	35 (47.9)	18 (47.4)	17 (48.6)
E19del	19 (26.0)	12 (31.6)	7 (20.0)
L858R	19 (26.0)	8 (21.1)	11 (31.4)

Note: Early stage refers to stage I - IIIA, and advanced stage refers to stage IIIB - IV

Abbreviation: E19del, (c.2235del15; p.E746_A750del); L858R, (c.2573 T > G; p.Leu858Arg)

### Prediction of tissue EGFR mutations using plasma and BWF ddPCR

**Fig. 1:**
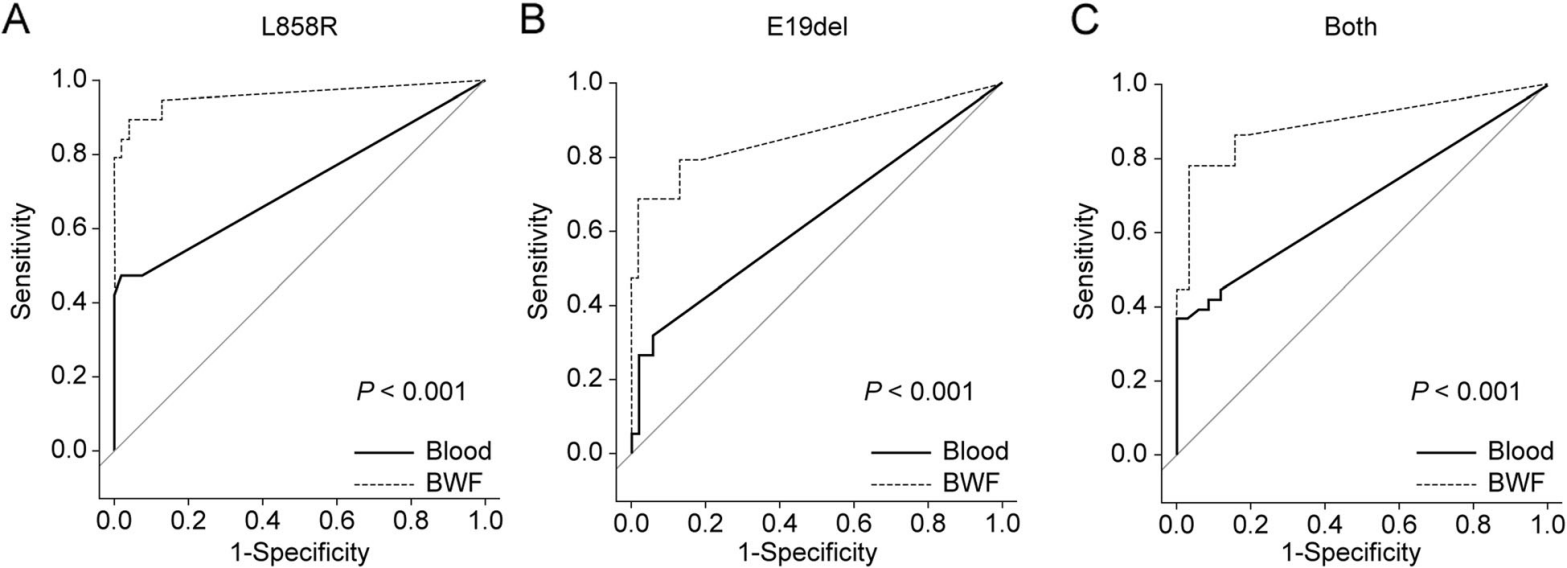
Comparison of receiver operator characteristic curves according to the sample type (plasma or BWF) and EGFR mutation genotype. **A** L858R; **B** E19del; and **C** both L858R and E19del. BWF, bronchial washing fluid. Abbreviation: E19del, (c.2235del15; p.E746_A750del); L858R, (c.2573 T > G; p.Leu858Arg).

First, we compared the diagnostic yields of plasma and BWF ddPCR for all cases. The AUCs were 0.717 [95% confidence interval (CI): 0.592–0.842] for L858R detection in the plasma samples and 0.961 (95% CI: 0.901–1.000) for that in the BWF samples (Table 2 and Fig. 1A). Thus, the testing of BWF more accurately predicted the presence of L858R in tumor tissue than did that of the plasma, and the difference between the AUCs was significant (*p* < 0.001), based on DeLong’s test for two correlated receiver operator characteristic (ROC) curves. Similar results were obtained for predicting the presence of E19del in tumor tissue, with the AUC being only 0.632 (95% CI: 0.519–0.745) for plasma ddPCR and 0.858 (95% CI: 0.746–0.969) for BWF ddPCR (Table 2 and Fig. 1B). Thus, BWF was also more useful than the plasma for predicting E19del, and the difference between the AUCs was significant (*p* < 0.001), as determined by DeLong’s test for two correlated ROC curves.

**Table 2:** Sensitivity, specificity, and concordance rate of ddPCR according to EGFR mutation

		L858R			E19del	
	Plasma	BWF	p-value	Plasma	BWF	p-value
AUC	0.717 (0.592–0.842)	0.961 (0.901–1.0)	< 0.001	0.632 (0.519–0.745)	0.858 (0.746–0.969)	< 0.001
Sensitivity (%)	47.37	89.47		31.58	68.42	
Specificity (%)	98.15	96.30		94.44	98.15	
Concordance rate	82.2% (60/73)	94.5% (69/73)		76.7% (56/73)	84.9% (62/73)	

Abbreviation: BWF, bronchial washing fluid; AUC, area under curve; E19del, (c.2235del15; p.E746_A750del); L858R, (c.2573 T>; p.Leu858Arg)

**Fig. 2:**
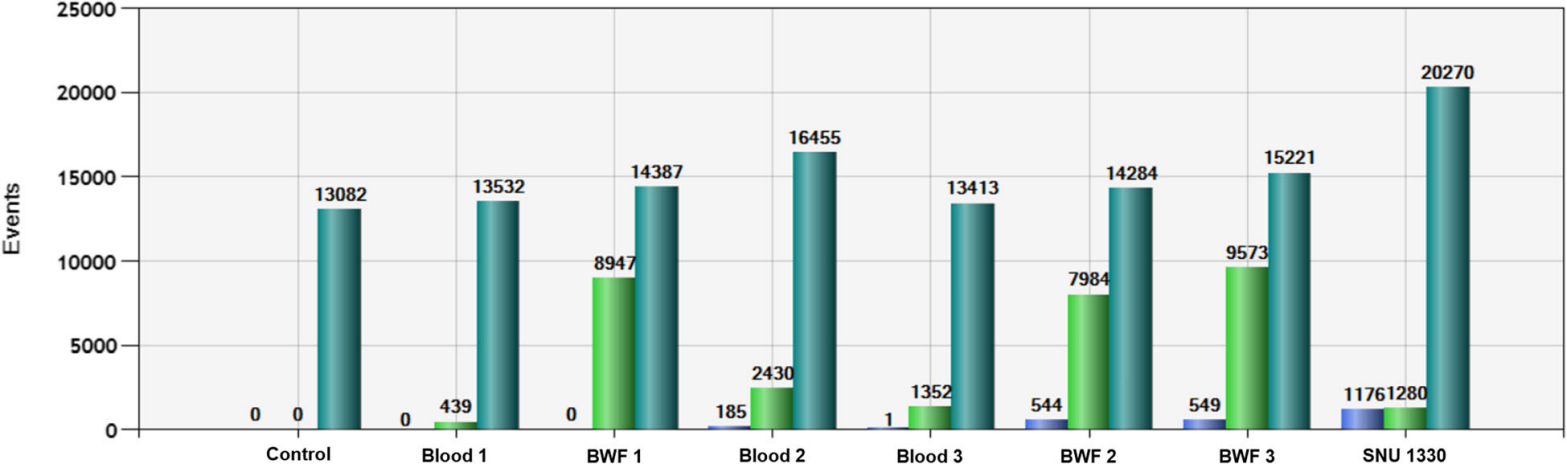
Example of differences in ddPCR results according to samples. Bronchial washing fluid (BWF 2 and 3) specimens showed clearly distinguishable mutational droplets from those in plasma (Plasma 2 and 3) specimens. SNU 1330 is the positive control. Blue bars indicate FAM-positive droplets, and green bars indicate HEX-positive droplets. Dark green bars indicate total droplets

By combining the results of L858R and E19del detection, variables were simplified, and the usefulness of the plasma and BWF was compared for predicting the tissue EGFR mutation status by ddPCR. The AUC for detecting tumor EGFR mutations using the plasma samples was 0.686 (95% CI: 0.592–0.780), whereas that using the BWF samples was 0.895 (95% CI: 0.822–0.969), showing a significant difference between the types of specimens (*p* < 0.0001; DeLong’s test for two correlated ROC curves). Comparison of the Fa value obtained from BWF with that obtained from the plasma for each patient showed that the former was significantly higher than the latter in mEGFR-positive cases (*p* = 0.004; Wilcoxon signed-rank test), indicating that BWF is more suitable than plasma to easily distinguish the positivity or negativity of ddPCR results (Fig. 2).

### Sensitivity of ddPCR for prediction of the EGFR mutation status

**Fig. 3:**
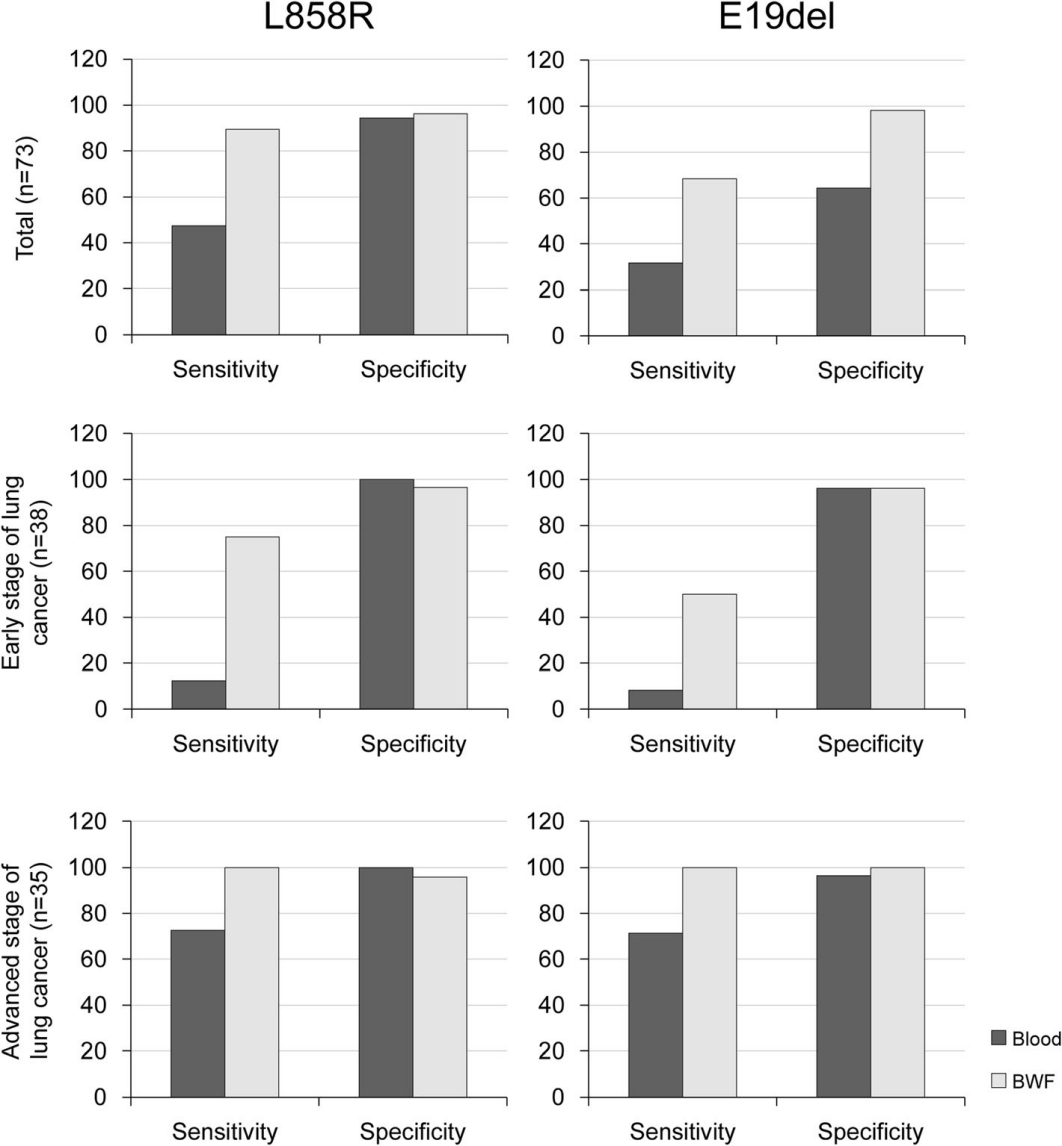
Comparison of sensitivity and specificity of ddPCR according to the EGFR mutation genotype and lung cancer stage. **A** Total patients; **B** patients with early-stage (I–IIIA) lung cancer; and **C** patients with advanced-stage (IIIB–IV) lung cancer. BWF, bronchial washing fluid. Abbreviation: E19del, (c.2235del15; p.E746_A750del); L858R, (c.2573 T > G; p.Leu858Arg).

The cutoff Fa value based on the results obtained for the plasma samples was 0.044%, and that for the BWF samples was 0.015%. When these cutoff values were applied, the sensitivity for predicting tissue E19del mutations using the plasma samples was 31.6%, whereas that using BWF was 68.4% (Fig. 3), demonstrating that BWF was superior (*p* = 0.005; McNemar’s chi-squared test). Similar results were obtained when predicting tumor tissue L858R substitution; the sensitivity using the plasma samples was 47.4%, while that using BWF was 89.5% (*p* = 0.005; McNemar’s chi-squared test). The same findings were obtained when comparing the usefulness of the plasma and BWF in predicting the tissue EGFR mutation status by combining the L858R and E19del values in one variable (*p* < 0.0001; McNemar’s chi-squared test). The results obtained using both types of specimens showed a high specificity, with no significant difference observed (data not shown).

### BWF ddPCR showed good diagnostic yields

The detection rate of mEGFRs using the plasma samples was dependent on the disease stage. To confirm whether the same findings can be obtained using BWF, we investigated the mEGFR detection yields in each sample type by dividing lung cancer into early and advanced stages and then compared the detection rates for each type of samples in each group (Table 3 and Fig. 3). In the early-stage group (stages I–IIIA; *n* = 38), the AUC value of the plasma samples for predicting tissue mEGFRs was 0.504, whereas that of BWF was 0.768, showing a significant difference between the sample types (*p* = 0.008). Although the specificity was high for both sample types, the sensitivity was only 15.0% for plasma ddPCR, while that for BWF ddPCR was 65.0%. In the advanced-stage group (stages IIIB–IV; *n* = 35), the AUC value obtained using BWF (1.000; 95% CI: 0.899–1.000) was higher than that obtained using the plasma (0.879; 95% CI: 0.724–0.964). These findings showed that the results obtained using BWF predicted the tumor tissue mEGFR status more accurately than did those obtained using plasma (*p* = 0.043; DeLong’s test for two correlated ROC curves).

**Table 3:** Sensitivity, specificity, and concordance rate of ddPCR according to lung cancer stage

	Early stage of lung cancer (n = 38)			Advanced stage of lung cancer (n = 35)		
	Plasma	BWF	p-value	Plasma	BWF	p-value
AUC	0.504 (0.338–0.670)	0.768 (0.603–0.889)	0.008	0.879 (0.724–0.964)	1.0 (0.899–1.000)	0.043
Sensitivity (%)	15.0	65.0		72.2	100.0	
Specificity (%)	100.0	88.9		100.0	100.0	

Note: Early stage refers to stage I - IIIA, and advanced stage refers to stage IIIB - IV

Abbreviation: BWF, bronchial washing fluid; AUC, area under curve

The plasma and BWF data were highly specific; however, the sensitivity of BWF ddPCR was significantly higher than that of plasma ddPCR. The values obtained using BWF also accurately reflected the mEGFR status in early- and advanced-stage tumors.

## Discussion

This study showed that BWF liquid biopsy samples more reliably reflected the tumor mEGFR status than did plasma samples when using ddPCR. Additionally, BWF could be used to analyze both L858R and E19del mutations and produced more sensitive results than did plasma at different stages of lung cancer.

Although tissue biopsy is the gold standard for molecular genotyping in lung cancer, liquid biopsy may play an important role as a complementary method for targeted gene detection and the prediction of the clinical course or outcome [15, 16], as well as for the detection of lung cancer at an early stage [17]. Liquid biopsy is a relatively non-invasive, safe, and simple procedure. Therefore, it can be performed in patients with poor medical conditions or when the location or size of lung cancer makes biopsy difficult.

Many studies have shown the feasibility of liquid biopsy for lung cancer, but some limitations remain [13, 18]. Blood samples are mainly used for liquid biopsy, including determination of ctDNA, circulating tumor cells, platelets, exosomes, and microRNAs, which provide specific genetic information on the tumor. Although the proportion of ctDNA can vary depending on the tumor burden, stage, vascularization, and biological features, the value is generally only 0.1–1.0%. Furthermore, ctDNA has a relatively short half-life, from approximately 16 to 150 min [19]. Thus, if sample processing is delayed, the experimental results would not reflect the precise ctDNA level. To overcome this limitation, we froze BWF and plasma within 3 and 8 h of collecting samples, respectively, and used Streck tubes, which maintain the stability of cfDNA for up to 14 days and CTC for up to 7 days. Recently, Krug el al [20]. demonstrated that compared with that of ctDNA, combined use of exosomal RNA (exoRNA) and cfDNA allowed the detection of activating EGFR mutations and T790M mutation with improved sensitivity (96 and 90%, respectively). In particular, this approach resulted in the largest improvement in sensitivity (26 to 74%) in patients with intrathoracic metastatic diseases. Therefore, measuring exoRNA and ctDNA in BWF specimens may greatly improve the detection of EGFR mutations, even at an early stage of lung cancer; however, further studies are needed to evaluate this possibility.

BAL plays a supporting role in the diagnosis of lung cancer [21]. Since the 1980s, various studies have shown the usefulness of BAL in the diagnosis of lung malignancies [22]. BAL showed a diagnostic yield of 33–90% in diffuse malignant pulmonary infiltrates, although the value differed depending on the cancer type. In particular, in NSCLC, such as squamous cell carcinoma and adenocarcinoma, the diagnostic yield was 50 and 77%, respectively [22]. Park et al. [23] suggested that BALF might be effective for determining the EGFR mutation status. Although their study involved a small number of subjects (*n* = 20), a high concordance rate (91.7%) was observed between BALF and tissue for detecting EGFR mutations using PNA-mediated PCR clamping and PANAMutyper with fluorescence melting curve analysis. However, there were only three patients with early-stage lung cancer, and they did not show any difference in the detection rate between BALF and plasma. Our study included 38 patients with an early-stage lung cancer, and the sensitivity of mEGFR detection was 65% using BWF and only 15.0% using plasma. The use of BWF showed great improvements in the sensitivity and diagnostic yield compared with that of the plasma in the case of patients with an early stage of lung cancer. Therefore, we suggest that BWF rather than plasma be used to detect mutational variations, regardless of the lung cancer stage.

There are various methods of mEGFR detection in lung cancer using liquid biopsy samples. ddPCR, which is based on the generation of ~ 20,000 droplets, is one of the powerful advanced techniques for detecting rare gene mutations. Sacher el al [24]. reported a sensitivity of 82 and 74% in the detection of E19del and L858R mutations, respectively, via ddPCR using liquid biopsy samples from patients with advanced lung cancer, and Thress el al [25]. reported a sensitivity of 90% and a specificity 100% in the case of the L858R mutation. In our study, ddPCR also showed high sensitivity and specificity using both plasma and BWF for the detection of mEGFRs in advanced-stage cancer.

Our study showed that BWF could be substituted for tissue biopsy samples to confirm EGFR mutations, and the use of BWF may shorten the time from the diagnosis to treatment by avoiding delays for biopsy and confirmation of biopsy results. BWF specimen collection is easier and safer in comparison with lung biopsy because only simple bronchoscopy needs to be performed in the former, for which hospitalization is not required.

Additionally, we investigated the relationship between tumor size and the mEGFR detection rate. Although a significant agreement between liquid biopsy and tissue biopsy samples was expected regarding the mEGFR status for larger-sized tumors, no significant association was noted between the tumor size and mEGFR detection rate for liquid biopsy and tissue biopsy samples (data not shown). These findings suggest that the diagnostic yield of ddPCR using BWF liquid biopsy samples mainly depends on the disease stage rather than on tumor size. However, further studies with larger sample sizes are needed.

## Conclusions

Compared with liquid biopsy using plasma, that using BWF resulted in a more effective detection of mEGFRs by ddPCR. Thus, BWF may be useful for avoiding invasive tissue biopsy and associated complications, such as pneumothorax or bleeding.

## Supplementary information

**Additional file 1.** Results for ddPCR experiments.

## Data Availability

The datasets used and/or analyzed during the current study are available in Additional file 1.

## Abbreviations

ctDNA: Circulating tumor DNA
mEGFR: EGFR-tyrosine kinase sensitizing mutation
ddPCR: Digital droplet PCR
AUC: Area under the curve
TKI: Tyrosine kinase inhibitor
cfDNA: Cell free DNA
BWF: Bronchial washing fluid
BALF: Bronchoalveolar lavage fluid

## References

[1] Siegel RL, Miller KD, Jemal A (2018). Cancer statistics, 2018. CA Cancer J Clin.

[2] de Groot PM, Wu CC, Carter BW, Munden RF (2018). The epidemiology of lung cancer. Transl Lung Cancer Res.

[3] Chan BA, Hughes BG (2015). Targeted therapy for non-small cell lung cancer: current standards and the promise of the future. Transl Lung Cancer Res.

[4] Thatcher N, Chang A, Parikh P, Rodrigues Pereira J, Ciuleanu T, von Pawel J, Thongprasert S, Tan EH, Pemberton K, Archer V, Carroll K (2005). Gefitinib plus best supportive care in previously treated patients with refractory advanced non-small-cell lung cancer: results from a randomised, placebo-controlled, multicentre study (Iressa survival evaluation in lung Cancer). Lancet.

[5] Lynch TJ, Bell DW, Sordella R, Gurubhagavatula S, Okimoto RA, Brannigan BW, Harris PL, Haserlat SM, Supko JG, Haluska FG (2004). Activating mutations in the epidermal growth factor receptor underlying responsiveness of non-small-cell lung cancer to gefitinib. N Engl J Med.

[6] Mok TS, Wu YL, Thongprasert S, Yang CH, Chu DT, Saijo N, Sunpaweravong P, Han B, Margono B, Ichinose Y (2009). Gefitinib or carboplatin-paclitaxel in pulmonary adenocarcinoma. N Engl J Med.

[7] Gonzalez-Larriba JL, Lazaro-Quintela M, Cobo M, Domine M, Majem M, Garcia-Campelo R (2017). Clinical management of epidermal growth factor receptor mutation-positive non-small cell lung cancer patients after progression on previous epidermal growth factor receptor tyrosine kinase inhibitors: the necessity of repeated molecular analysis. Transl Lung Cancer Res.

[8] Rivera MP, Mehta AC, Wahidi MM (2013). Establishing the diagnosis of lung cancer: diagnosis and management of lung cancer, 3rd ed: American College of Chest Physicians evidence-based clinical practice guidelines. Chest.

[9] Tam AL, Kim ES, Lee JJ, Ensor JE, Hicks ME, Tang X, Blumenschein GR, Alden CM, Erasmus JJ, Tsao A (2013). Feasibility of image-guided transthoracic core-needle biopsy in the BATTLE lung trial. J Thorac Oncol.

[10] Diaz LA, Bardelli A (2014). Liquid biopsies: genotyping circulating tumor DNA. J Clin Oncol.

[11] Oumeraci T, Schmidt B, Wolf T, Zapatka M, Pich A, Brors B, Eils R, Fleischhacker M, Schlegelberger B, von Neuhoff N (2011). Bronchoalveolar lavage fluid of lung cancer patients: mapping the uncharted waters using proteomics technology. Lung Cancer.

[12] Carvalho AS, Cuco CM, Lavareda C, Miguel F, Ventura M, Almeida S, Pinto P, de Abreu TT, Rodrigues LV, Seixas S (2017). Bronchoalveolar lavage proteomics in patients with suspected lung Cancer. Sci Rep.

[13] Lim M, Kim CJ, Sunkara V, Kim MH, Cho YK. Liquid biopsy in lung Cancer: clinical applications of circulating biomarkers (CTCs and ctDNA). Micromachines (Basel). 2018;9. 10.3390/mi9030100 PMC6187707 30424034

[14] Feng WN, Gu WQ, Zhao N, Pan YM, Luo W, Zhang H, Liang JM, Yang J, Deng YM (2018). Comparison of the SuperARMS and droplet digital PCR for detecting EGFR mutation in ctDNA from NSCLC patients. Transl Oncol.

[15] Luo W, Rao M, Qu J, Luo D (2018). Applications of liquid biopsy in lung cancer-diagnosis, prognosis prediction, and disease monitoring. Am J Transl Res.

[16] Hong MH, Kim HR, Ahn BC, Heo SJ, Kim JH, Cho BC (2019). Real-world analysis of the efficacy of Rebiopsy and EGFR mutation test of tissue and plasma samples in drug-resistant non-small cell lung Cancer. Yonsei Med J.

[17] Liang W, Zhao Y, Huang W, Liang H, Zeng H, He J (2018). Liquid biopsy for early stage lung cancer. J Thorac Dis.

[18] Cabanero M, Tsao MS (2018). Circulating tumour DNA in EGFR-mutant non-small-cell lung cancer. Curr Oncol.

[19] Saarenheimo J, Eigeliene N, Andersen H, Tiirola M, Jekunen A (2019). The value of liquid biopsies for guiding therapy decisions in non-small cell lung Cancer. Front Oncol.

[20] Krug AK, Enderle D, Karlovich C, Priewasser T, Bentink S, Spiel A, Brinkmann K, Emenegger J, Grimm DG, Castellanos-Rizaldos E (2018). Improved EGFR mutation detection using combined exosomal RNA and circulating tumor DNA in NSCLC patient plasma. Ann Oncol.

[21] Semenzato G, Poletti V (1992). Bronchoalveolar lavage in lung cancer. Respiration.

[22] Poletti V, Poletti G, Murer B, Saragoni L, Chilosi M (2007). Bronchoalveolar lavage in malignancy. Semin Respir Crit Care Med.

[23] Park S, Hur JY, Lee KY, Lee JC, Rho JK, Shin SH, Choi CM (2017). Assessment of EGFR mutation status using cell-free DNA from bronchoalveolar lavage fluid. Clin Chem Lab Med.

[24] Sacher AG, Paweletz C, Dahlberg SE, Alden RS, O'Connell A, Feeney N, Mach SL, Janne PA, Oxnard GR (2016). Prospective validation of rapid plasma genotyping for the detection of EGFR and KRAS mutations in advanced lung Cancer. JAMA Oncol.

[25] Thress KS, Brant R, Carr TH, Dearden S, Jenkins S, Brown H, Hammett T, Cantarini M, Barrett JC (2015). EGFR mutation detection in ctDNA from NSCLC patient plasma: a cross-platform comparison of leading technologies to support the clinical development of AZD9291. Lung Cancer.

